# Chlorido(1,3-dimethyl­thio­urea-κ*S*)bis­(triphenyl­phosphine-κ*P*)copper(I) acetonitrile hemisolvate

**DOI:** 10.1107/S1600536809026798

**Published:** 2009-07-15

**Authors:** Latipah La-o, Chaveng Pakawatchai, Saowanit Saithong, Brian W. Skelton

**Affiliations:** a Department of Chemistry, Faculty of Science, Prince of Songkla University, Hat Yai, Songkhla 90112, Thailand; b School of Biomedical, Biomolecular and Chemical Sciences, University of Western Australia, Crawley, Western Australia 6009, Australia

## Abstract

The title compound, [CuCl(C_3_H_8_N_2_S)(C_18_H_15_P)_2_]·0.5CH_3_CN, was prepared by the reaction of copper(I) chloride with 1,3-dimethyl­thio­urea (dmtu) and triphenyl­phosphine (PPh_3_) in acetonitrile. The Cu^I^ atom has a distorted tetra­hedral environment formed by two P atoms from triphenyl­phosphine, one S atom from the dmtu ligand and one Cl atom. In addition, the mol­ecules exhibit intra- and inter­molecular N—H⋯Cl inter­actions.

## Related literature

For related structures, see: Aslanidis *et al.* (1993 ▶, 1998 ▶); Cox *et al.* (1999 ▶); Karagiannidis *et al.* (1990 ▶); Lecomte *et al.* (1989 ▶); Singh & Dikshit (1995 ▶); Skoulika *et al.* (1991 ▶).
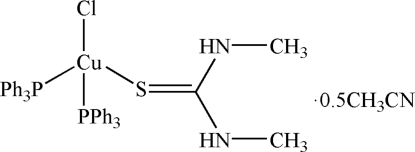


## Experimental

### 

#### Crystal data

[CuCl(C_3_H_8_N_2_S)(C_18_H_15_P)_2_]·0.5C_2_H_3_N
*M* *_r_* = 748.23
Monoclinic, 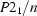

*a* = 13.7503 (4) Å
*b* = 30.0495 (9) Å
*c* = 18.4227 (5) Å
β = 90.874 (1)°
*V* = 7611.2 (4) Å^3^
*Z* = 8
Mo *K*α radiation
μ = 0.81 mm^−1^
*T* = 293 K
0.36 × 0.12 × 0.08 mm

#### Data collection

Bruker SMART CCD area-detector diffractometer
Absorption correction: multi-scan (*SADABS*; Bruker, 2003 ▶) *T*_min_ = 0.777, *T*_max_ = 0.940
70507 measured reflections
13413 independent reflections
10371 reflections with *I* > 2s(*I*)
*R*_int_ = 0.059

#### Refinement

*R*[*F* ^2^ > 2σ(*F* ^2^)] = 0.053
*wR*(*F* ^2^) = 0.111
*S* = 1.10
13413 reflections
873 parameters
4 restraints
H atoms treated by a mixture of independent and constrained refinement
Δρ_max_ = 0.53 e Å^−3^
Δρ_min_ = −0.25 e Å^−3^

### 

Data collection: *SMART* (Bruker, 1998 ▶); cell refinement: *SAINT* (Bruker, 1998 ▶); data reduction: *SAINT*; program(s) used to solve structure: *SHELXS97* (Sheldrick, 2008 ▶); program(s) used to refine structure: *SHELXL97* (Sheldrick, 2008 ▶); molecular graphics: *SHELXTL* (Sheldrick, 2008 ▶); software used to prepare material for publication: *SHELXTL*.

## Supplementary Material

Crystal structure: contains datablocks I, global. DOI: 10.1107/S1600536809026798/pk2174sup1.cif

Structure factors: contains datablocks I. DOI: 10.1107/S1600536809026798/pk2174Isup2.hkl

Additional supplementary materials:  crystallographic information; 3D view; checkCIF report

## Figures and Tables

**Table 1:** Hydrogen-bond geometry (Å, °)

*D*—H⋯*A*	*D*—H	H⋯*A*	*D*⋯*A*	*D*—H⋯*A*
N1*A*—H1*AA*⋯Cl1*B*^i^	0.875 (18)	2.43 (2)	3.234 (3)	153 (3)
N2*A*—H2*AA*⋯Cl1*A*	0.875 (18)	2.326 (19)	3.197 (3)	173 (3)
N1*B*—H1*BB*⋯Cl1*A*	0.869 (18)	2.47 (2)	3.262 (3)	152 (3)
N2*B*—H2*BB*⋯Cl1*B*	0.879 (18)	2.36 (2)	3.230 (3)	169 (3)

Symmetry code: (i) 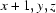.

**Fig. 1.**
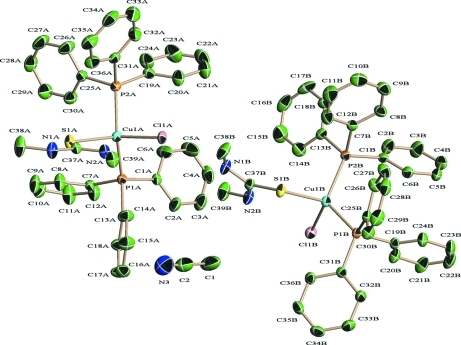
The molecular structure of the [CuCl(dmtu)(PPh3)2].0.5CH3CN complex. Thermal ellipsoids are shown at the 25% probability level.

## supplementary crystallographic information

### Comment

Treatment of [Cu(PPh_3_)_3_Cl] (PPh_3 _= triphenylphosphine) in acetonitrile with *N,N*'-dimethylthiourea(dmtu) in a 1:2 metal-thione ratio yielded a product of formula [Cu(PPh_3_)_2_(dmtu)Cl], 0.5 CH_3_CN. Its crystal structure consists of two independent [CuCl(PPh_3_)_2_(dmtu)] molecules (A and B) plus a CH_3_CN solvent molecule in the asymmetric unit. The Cu^I^ atoms display a distorted tetrahedral environment (Fig. 1). Distorted tetrahedral geometries are also found in similar phosphine adducts of Cu^I^ halides (Aslanidis *et al.*, 1993, 1998; Cox *et al.*, 1999; Karagiannidis *et al.*, 1990; Lecomte *et al., *1989; Singh & Dikshit, 1995; Skoulika *et al.*, 1991). In both A and B molecules the distorted tetrahedral coordination consists of the S donor of the *N,N*'-dimethylthiourea ligand, two P atoms of two phosphine ligands, as well as the Cl atom. The Cu—P(1) and Cu—P(2) distances of 2.2847 (9), 2.2850 (9) Å and 2.2831 (9), 2.2989 (9) Å in molecule A and B, are slightly shorter than the corresponding lengths observed in [Cu(PPh_3_)_2_(tzdtH)Cl](Aslanidis *et al., *1998). The P(1)—Cu—P(2) angle deviates considerably from the ideal tetrahedral value of 109.4° [124.71 (4)° (A) and 120.07 (3)° (B)]. These values are more similar to those found in trigonally coordinated Cu^I^, a mode which is essentially determined by steric bulky ligands and by constraints related to intra-molecular hydrogen bridging bonds. Other significant features of the present structure are the Cu—S and Cu—Cl bond lengths which lie in the range normally observed for tetrahedrally coordinated Cu^I^ complexes with terminal chloride and thione-sulfur donors. The Cu—S bond lengths [2.3715 (10) (A) 2.3857 (9) Å (B)] are longer than in [Cu(PPh_3_)_2_(pymtH)I][2.338 (4) Å] (Aslanidis *et al.*, 1993) but shorter than in [Cu(PPh_3_)_2_ (tzdtH)Cl] [2.418 (5) Å] (Aslanidis *et al.*, 1998). The observed Cu—Cl distances of 2.4014 (9), 2.3956 (9) Å in molecule A and B, respectively, are very close to those observed in [Cu(PPh_3_)_2_(bztzdtH)Cl] [2.40 (2) Å] (Cox *et al.*, 1999). In both molecules, the Cl atom is hydrogen bonded to the *N,N*'-dimethylthiourea NH atoms as shown in Table 1. These hydrogen bonds may be the main reason for the conformational changes, *i.e.* the increase of the P—Cu—P angle and distortion from the tetrahedral configuration. This hydrogen bonding may also influence the orientation of the complexed ligands (Skoulika *et al.*, 1991).

### Experimental

Triphenylphosphine was added to an acetonitrile suspension of Cu^I^ chloride. After stirring for 2 h, *N,N*'-dimethylthiourea was added. The mixture was refluxed for 5 h to afford a colorless solution. Single crystals were obtained after cooling followed by slow evaporation overnight at room temperature. The melting point of the complex is 469–470 K. Elemental analysis, calculated for [CuCl(dmtu)(PPh_3_)_2_], 0.5 CH_3_CN: C, 64.37; H, 5.26; N, 3.85; S,4.40%, found: C, 64.54; H, 5.58; N, 3.70; S, 4.52%.

### Refinement

The structure was solved by direct methods and refined by full-matrix least-squares procedure based on *F^2^*. The hydrogen atoms of the amine N were located in a difference Fourier map and refined with geometrical restraints [N—H = 0.87–0.89 Å and *U*_iso_(H) = 1.2*U*_eq_(N)]. All C Hydrogen atoms were placed in geometrically idealized positions and refined isotropically with a riding model for both C-*sp*^2^ [C—H = 0.93 Å and with *U*_iso_(H) = 1.2*U*_eq_(C)] and C-*sp*^3^ [C—H = 0.96 Å and with *U*_iso_(H) = 1.5*U*_eq_(C)].

### Crystal data

**Table:**

[CuCl(C_3_H_8_N_2_S)(C_18_H_15_P)_2_]·0.5C_2_H_3_N	*F*(000) = 3112
*M**_r_* = 748.23	*D*_x_ = 1.306 Mg m^−^^3^
Monoclinic, *P*2_1_/*n*	Mo *K*α radiation, λ = 0.71073 Å
Hall symbol: -P 2yn	Cell parameters from 7629 reflections
*a* = 13.7503 (4) Å	θ = 2.2–21.5°
*b* = 30.0495 (9) Å	µ = 0.81 mm^−^^1^
*c* = 18.4227 (5) Å	*T* = 293 K
β = 90.874 (1)°	Block, colorless
*V* = 7611.2 (4) Å^3^	0.36 × 0.12 × 0.08 mm
*Z* = 8	

### Data collection

**Table:**

Bruker SMART CCD area-detector diffractometer	13413 independent reflections
Radiation source: fine-focus sealed tube	10371 reflections with *I* > 2s(*I*)
graphite	*R*_int_ = 0.059
Frames each covering 0.3 ° in ω scans	θ_max_ = 25.0°, θ_min_ = 1.3°
Absorption correction: multi-scan (*SADABS*; Bruker, 2003)	*h* = −16→16
*T*_min_ = 0.777, *T*_max_ = 0.940	*k* = −35→35
70507 measured reflections	*l* = −21→21

### Refinement

**Table:**

Refinement on *F*^2^	Primary atom site location: structure-invariant direct methods
Least-squares matrix: full	Secondary atom site location: difference Fourier map
*R*[*F*^2^ > 2σ(*F*^2^)] = 0.053	Hydrogen site location: inferred from neighbouring sites
*wR*(*F*^2^) = 0.111	H atoms treated by a mixture of independent and constrained refinement
*S* = 1.10	*w* = 1/[σ^2^(*F*_o_^2^) + (0.0428*P*)^2^ + 3.4594*P*] where *P* = (*F*_o_^2^ + 2*F*_c_^2^)/3
13413 reflections	(Δ/σ)_max_ = 0.001
873 parameters	Δρ_max_ = 0.53 e Å^−^^3^
4 restraints	Δρ_min_ = −0.24 e Å^−^^3^

### Special details

**Table:**

Geometry. All e.s.d.'s (except the e.s.d. in the dihedral angle between two l.s. planes) are estimated using the full covariance matrix. The cell e.s.d.'s are taken into account individually in the estimation of e.s.d.'s in distances, angles and torsion angles; correlations between e.s.d.'s in cell parameters are only used when they are defined by crystal symmetry. An approximate (isotropic) treatment of cell e.s.d.'s is used for estimating e.s.d.'s involving l.s. planes.
Refinement. Refinement of *F*^2^ against ALL reflections. The weighted *R*-factor *wR* and goodness of fit *S* are based on *F*^2^, conventional *R*-factors *R* are based on *F*, with *F* set to zero for negative *F*^2^. The threshold expression of *F*^2^ > σ(*F*^2^) is used only for calculating *R*-factors(gt) *etc*. and is not relevant to the choice of reflections for refinement. *R*-factors based on *F*^2^ are statistically about twice as large as those based on *F*, and *R*- factors based on ALL data will be even larger.

### Fractional atomic coordinates and isotropic or equivalent isotropic displacement parameters (Å^2^)

**Table:**

	*x*	*y*	*z*	*U*_iso_*/*U*_eq_	
Cu1A	0.83366 (3)	0.723343 (13)	0.16892 (2)	0.03965 (11)	
Cl1A	0.76325 (6)	0.65021 (3)	0.16728 (5)	0.0516 (2)	
S1A	0.99851 (6)	0.72354 (3)	0.20869 (6)	0.0512 (2)	
N1A	1.1290 (2)	0.66507 (10)	0.2551 (2)	0.0614 (9)	
H1AA	1.148 (3)	0.6385 (8)	0.269 (2)	0.074*	
N2A	0.9764 (2)	0.63787 (9)	0.23609 (17)	0.0519 (8)	
H2AA	0.9202 (17)	0.6429 (12)	0.2147 (18)	0.062*	
P1A	0.74601 (6)	0.76174 (3)	0.25265 (5)	0.0380 (2)	
P2A	0.84109 (6)	0.74107 (3)	0.04852 (5)	0.0416 (2)	
C1A	0.6156 (2)	0.76738 (11)	0.23128 (19)	0.0427 (8)	
C2A	0.5422 (3)	0.76381 (15)	0.2798 (2)	0.0726 (12)	
H2A	0.5568	0.7575	0.3282	0.087*	
C3A	0.4458 (3)	0.76946 (18)	0.2580 (3)	0.0923 (16)	
H3A	0.3968	0.7674	0.2921	0.111*	
C4A	0.4228 (3)	0.77790 (16)	0.1880 (3)	0.0861 (15)	
H4A	0.3581	0.7813	0.1736	0.103*	
C5A	0.4942 (4)	0.7813 (2)	0.1390 (3)	0.114 (2)	
H5A	0.4792	0.7872	0.0906	0.137*	
C6A	0.5902 (3)	0.77597 (19)	0.1611 (2)	0.0923 (17)	
H6A	0.6389	0.7783	0.1269	0.111*	
C7A	0.7845 (2)	0.81944 (10)	0.26739 (17)	0.0401 (8)	
C8A	0.8823 (3)	0.82841 (13)	0.2747 (3)	0.0724 (13)	
H8A	0.9263	0.8049	0.2757	0.087*	
C9A	0.9167 (3)	0.87141 (14)	0.2805 (3)	0.0850 (15)	
H9A	0.9833	0.8765	0.2847	0.102*	
C10A	0.8543 (3)	0.90602 (13)	0.2802 (3)	0.0751 (13)	
H10A	0.8777	0.9350	0.2819	0.090*	
C11A	0.7566 (3)	0.89813 (13)	0.2774 (3)	0.0860 (15)	
H11A	0.7131	0.9218	0.2800	0.103*	
C12A	0.7222 (3)	0.85525 (12)	0.2706 (2)	0.0687 (12)	
H12A	0.6554	0.8504	0.2681	0.082*	
C13A	0.7527 (2)	0.73857 (11)	0.34471 (17)	0.0426 (8)	
C14A	0.7917 (3)	0.69658 (13)	0.3537 (2)	0.0622 (11)	
H14A	0.8113	0.6805	0.3134	0.075*	
C15A	0.8018 (4)	0.67813 (15)	0.4224 (2)	0.0804 (14)	
H15A	0.8288	0.6499	0.4279	0.096*	
C16A	0.7725 (4)	0.70115 (17)	0.4815 (2)	0.0815 (14)	
H16A	0.7785	0.6885	0.5274	0.098*	
C17A	0.7343 (4)	0.74264 (16)	0.4739 (2)	0.0836 (15)	
H17A	0.7143	0.7583	0.5146	0.100*	
C18A	0.7252 (3)	0.76151 (13)	0.4055 (2)	0.0680 (12)	
H18A	0.7001	0.7901	0.4007	0.082*	
C19A	0.7252 (3)	0.74469 (12)	−0.00126 (18)	0.0502 (9)	
C20A	0.6629 (3)	0.70896 (15)	0.0034 (2)	0.0736 (12)	
H20A	0.6815	0.6840	0.0301	0.088*	
C21A	0.5732 (3)	0.70979 (19)	−0.0312 (3)	0.0947 (16)	
H21A	0.5330	0.6849	−0.0290	0.114*	
C22A	0.5429 (4)	0.7460 (2)	−0.0681 (3)	0.0972 (17)	
H22A	0.4820	0.7464	−0.0907	0.117*	
C23A	0.6016 (4)	0.7815 (2)	−0.0720 (3)	0.1074 (19)	
H23A	0.5806	0.8067	−0.0969	0.129*	
C24A	0.6937 (3)	0.78118 (16)	−0.0393 (2)	0.0827 (14)	
H24A	0.7340	0.8059	−0.0433	0.099*	
C25A	0.9008 (3)	0.79381 (11)	0.02850 (18)	0.0470 (8)	
C26A	0.9612 (4)	0.80061 (15)	−0.0296 (3)	0.0893 (16)	
H26A	0.9731	0.7775	−0.0619	0.107*	
C27A	1.0042 (4)	0.84182 (18)	−0.0398 (3)	0.1056 (19)	
H27A	1.0466	0.8456	−0.0782	0.127*	
C28A	0.9863 (4)	0.87628 (16)	0.0041 (3)	0.0877 (15)	
H28A	1.0132	0.9041	−0.0052	0.105*	
C29A	0.9285 (4)	0.87006 (15)	0.0622 (3)	0.0868 (15)	
H29A	0.9171	0.8935	0.0940	0.104*	
C30A	0.8865 (3)	0.82911 (13)	0.0743 (2)	0.0698 (12)	
H30A	0.8475	0.8253	0.1146	0.084*	
C31A	0.9103 (3)	0.70020 (11)	−0.00354 (19)	0.0490 (9)	
C32A	0.8768 (3)	0.67905 (16)	−0.0637 (3)	0.0822 (14)	
H32A	0.8151	0.6858	−0.0820	0.099*	
C33A	0.9334 (4)	0.64731 (18)	−0.0987 (3)	0.1049 (19)	
H33A	0.9094	0.6329	−0.1400	0.126*	
C34A	1.0232 (4)	0.63756 (15)	−0.0725 (3)	0.0922 (17)	
H34A	1.0608	0.6163	−0.0956	0.111*	
C35A	1.0585 (4)	0.65843 (16)	−0.0133 (3)	0.0821 (14)	
H35A	1.1210	0.6522	0.0038	0.099*	
C36A	1.0018 (3)	0.68913 (14)	0.0220 (2)	0.0679 (12)	
H36A	1.0258	0.7027	0.0640	0.082*	
C37A	1.0368 (2)	0.67178 (11)	0.23477 (18)	0.0433 (8)	
C38A	1.2041 (3)	0.69905 (14)	0.2544 (3)	0.0906 (16)	
H38D	1.2062	0.7125	0.2072	0.136*	
H38E	1.2660	0.6857	0.2657	0.136*	
H38F	1.1899	0.7214	0.2900	0.136*	
C39A	0.9991 (3)	0.59398 (12)	0.2634 (2)	0.0679 (11)	
H39A	1.0525	0.5818	0.2369	0.102*	
H39B	0.9433	0.5751	0.2575	0.102*	
H39C	1.0166	0.5959	0.3140	0.102*	
Cu1B	0.31137 (3)	0.519217 (13)	0.25982 (2)	0.03941 (11)	
Cl1B	0.26570 (6)	0.58658 (3)	0.31991 (5)	0.0490 (2)	
S1B	0.48449 (6)	0.51390 (3)	0.26225 (5)	0.0475 (2)	
N1B	0.6224 (2)	0.57018 (10)	0.22284 (19)	0.0599 (9)	
H1BB	0.646 (3)	0.5968 (8)	0.219 (2)	0.072*	
N2B	0.4872 (2)	0.60175 (10)	0.26940 (19)	0.0606 (9)	
H2BB	0.4301 (18)	0.5976 (13)	0.2892 (19)	0.073*	
P1B	0.25868 (6)	0.52429 (3)	0.14108 (4)	0.0389 (2)	
P2B	0.25668 (6)	0.46303 (3)	0.33194 (5)	0.0377 (2)	
C1B	0.1268 (2)	0.52181 (11)	0.12563 (17)	0.0412 (8)	
C2B	0.0757 (3)	0.55385 (15)	0.0873 (2)	0.0701 (12)	
H2B	0.1085	0.5779	0.0674	0.084*	
C3B	−0.0243 (3)	0.55041 (19)	0.0783 (3)	0.0877 (15)	
H3B	−0.0581	0.5724	0.0530	0.105*	
C4B	−0.0733 (3)	0.51538 (17)	0.1060 (2)	0.0743 (13)	
H4B	−0.1401	0.5129	0.0985	0.089*	
C5B	−0.0240 (3)	0.48373 (15)	0.1452 (2)	0.0651 (11)	
H5B	−0.0576	0.4599	0.1651	0.078*	
C6B	0.0758 (3)	0.48702 (13)	0.15543 (19)	0.0523 (9)	
H6B	0.1086	0.4655	0.1827	0.063*	
C7B	0.3047 (2)	0.48048 (12)	0.08137 (18)	0.0453 (8)	
C8B	0.4042 (3)	0.47558 (15)	0.0777 (2)	0.0757 (13)	
H8B	0.4446	0.4937	0.1059	0.091*	
C9B	0.4443 (3)	0.44415 (17)	0.0327 (3)	0.0928 (16)	
H9B	0.5115	0.4416	0.0296	0.111*	
C10B	0.3848 (4)	0.41640 (16)	−0.0079 (2)	0.0827 (14)	
H10B	0.4118	0.3951	−0.0383	0.099*	
C11B	0.2867 (3)	0.42031 (15)	−0.0032 (2)	0.0730 (12)	
H11B	0.2463	0.4014	−0.0299	0.088*	
C12B	0.2472 (3)	0.45203 (13)	0.0408 (2)	0.0607 (10)	
H12B	0.1800	0.4544	0.0433	0.073*	
C13B	0.2930 (2)	0.57442 (12)	0.09057 (19)	0.0462 (9)	
C14B	0.3182 (3)	0.61196 (14)	0.1278 (2)	0.0780 (13)	
H14B	0.3179	0.6118	0.1783	0.094*	
C15B	0.3444 (4)	0.65045 (17)	0.0913 (3)	0.1007 (17)	
H15B	0.3627	0.6756	0.1175	0.121*	
C16B	0.3434 (3)	0.65169 (19)	0.0180 (3)	0.0911 (17)	
H16B	0.3598	0.6777	−0.0062	0.109*	
C17B	0.3186 (3)	0.6148 (2)	−0.0197 (3)	0.0870 (16)	
H17B	0.3184	0.6155	−0.0701	0.104*	
C18B	0.2935 (3)	0.57604 (15)	0.0156 (2)	0.0674 (11)	
H18B	0.2768	0.5509	−0.0112	0.081*	
C19B	0.1242 (2)	0.45675 (11)	0.33569 (18)	0.0439 (8)	
C20B	0.0712 (3)	0.49408 (13)	0.3533 (2)	0.0608 (10)	
H20B	0.1035	0.5208	0.3618	0.073*	
C21B	−0.0286 (3)	0.49246 (17)	0.3586 (3)	0.0784 (13)	
H21B	−0.0631	0.5178	0.3717	0.094*	
C22B	−0.0763 (3)	0.45380 (19)	0.3446 (3)	0.0845 (15)	
H22B	−0.1438	0.4529	0.3468	0.101*	
C23B	−0.0262 (3)	0.41626 (18)	0.3273 (3)	0.0834 (14)	
H23B	−0.0595	0.3898	0.3186	0.100*	
C24B	0.0752 (3)	0.41735 (13)	0.3228 (2)	0.0618 (11)	
H24B	0.1095	0.3917	0.3112	0.074*	
C25B	0.2967 (2)	0.40785 (11)	0.30464 (19)	0.0426 (8)	
C26B	0.2962 (3)	0.39879 (12)	0.2305 (2)	0.0580 (10)	
H26B	0.2783	0.4211	0.1979	0.070*	
C27B	0.3214 (3)	0.35760 (14)	0.2044 (2)	0.0688 (12)	
H27B	0.3191	0.3521	0.1548	0.083*	
C28B	0.3500 (3)	0.32469 (13)	0.2513 (3)	0.0704 (12)	
H28B	0.3673	0.2968	0.2336	0.084*	
C29B	0.3532 (3)	0.33287 (14)	0.3238 (3)	0.0729 (13)	
H29B	0.3736	0.3106	0.3556	0.088*	
C30B	0.3262 (3)	0.37405 (12)	0.3510 (2)	0.0588 (10)	
H30B	0.3281	0.3790	0.4008	0.071*	
C31B	0.2855 (2)	0.46715 (11)	0.42944 (17)	0.0418 (8)	
C32B	0.2338 (3)	0.44386 (13)	0.48077 (19)	0.0573 (10)	
H32B	0.1835	0.4250	0.4662	0.069*	
C33B	0.2566 (3)	0.44838 (15)	0.5535 (2)	0.0691 (12)	
H33B	0.2225	0.4320	0.5876	0.083*	
C34B	0.3290 (3)	0.47682 (15)	0.5759 (2)	0.0680 (12)	
H34B	0.3439	0.4799	0.6251	0.082*	
C35B	0.3792 (3)	0.50070 (14)	0.5254 (2)	0.0643 (11)	
H35B	0.4279	0.5203	0.5404	0.077*	
C36B	0.3578 (2)	0.49587 (12)	0.4522 (2)	0.0525 (9)	
H36B	0.3925	0.5121	0.4182	0.063*	
C37B	0.5344 (2)	0.56539 (11)	0.25007 (18)	0.0439 (8)	
C38B	0.6826 (3)	0.53423 (14)	0.1979 (3)	0.0823 (14)	
H38A	0.6986	0.5150	0.2379	0.123*	
H38B	0.7414	0.5461	0.1781	0.123*	
H38C	0.6482	0.5177	0.1611	0.123*	
C39B	0.5206 (3)	0.64687 (13)	0.2571 (3)	0.0872 (15)	
H39D	0.5827	0.6511	0.2806	0.131*	
H39E	0.4746	0.6675	0.2766	0.131*	
H39F	0.5266	0.6519	0.2059	0.131*	
C1	0.8259 (4)	0.4111 (3)	0.5006 (3)	0.139 (3)	
H1A	0.8137	0.4421	0.4925	0.208*	
H1B	0.7853	0.4006	0.5389	0.208*	
H1C	0.8115	0.3947	0.4570	0.208*	
C2	0.9262 (5)	0.4048 (2)	0.5207 (3)	0.1050 (19)	
N3	1.0045 (4)	0.3995 (2)	0.5354 (3)	0.137 (2)	

### Atomic displacement parameters (Å^2^)

**Table:**

	*U*^11^	*U*^22^	*U*^33^	*U*^12^	*U*^13^	*U*^23^
Cu1A	0.0382 (2)	0.0358 (2)	0.0451 (2)	0.00163 (18)	0.00370 (18)	0.00084 (18)
Cl1A	0.0468 (5)	0.0421 (5)	0.0659 (6)	−0.0130 (4)	0.0014 (4)	−0.0015 (4)
S1A	0.0393 (5)	0.0341 (5)	0.0800 (7)	0.0013 (4)	−0.0097 (4)	0.0030 (4)
N1A	0.0402 (18)	0.0399 (18)	0.104 (3)	0.0069 (15)	−0.0097 (17)	0.0051 (18)
N2A	0.0470 (18)	0.0355 (16)	0.073 (2)	0.0012 (15)	−0.0089 (16)	0.0043 (15)
P1A	0.0353 (5)	0.0359 (5)	0.0429 (5)	0.0026 (4)	0.0031 (4)	−0.0013 (4)
P2A	0.0406 (5)	0.0413 (5)	0.0431 (5)	0.0016 (4)	0.0042 (4)	0.0019 (4)
C1A	0.0356 (19)	0.0387 (19)	0.054 (2)	0.0011 (15)	0.0014 (16)	−0.0101 (16)
C2A	0.043 (2)	0.094 (3)	0.080 (3)	−0.006 (2)	0.009 (2)	0.009 (3)
C3A	0.043 (3)	0.118 (4)	0.116 (4)	−0.006 (3)	0.016 (3)	−0.003 (4)
C4A	0.041 (3)	0.094 (4)	0.122 (4)	0.009 (2)	−0.016 (3)	−0.028 (3)
C5A	0.065 (3)	0.202 (7)	0.074 (3)	0.040 (4)	−0.017 (3)	−0.023 (4)
C6A	0.045 (3)	0.169 (5)	0.062 (3)	0.031 (3)	0.000 (2)	−0.014 (3)
C7A	0.0387 (19)	0.0383 (18)	0.0432 (19)	0.0047 (15)	0.0003 (15)	−0.0016 (15)
C8A	0.044 (2)	0.048 (2)	0.125 (4)	0.0050 (19)	0.002 (2)	−0.029 (2)
C9A	0.045 (2)	0.066 (3)	0.144 (5)	−0.010 (2)	0.018 (3)	−0.037 (3)
C10A	0.077 (3)	0.041 (2)	0.108 (4)	−0.010 (2)	0.016 (3)	−0.002 (2)
C11A	0.072 (3)	0.038 (2)	0.148 (5)	0.008 (2)	−0.016 (3)	0.000 (3)
C12A	0.053 (2)	0.042 (2)	0.110 (3)	0.0044 (19)	−0.010 (2)	−0.002 (2)
C13A	0.044 (2)	0.0391 (19)	0.045 (2)	−0.0037 (16)	0.0027 (16)	0.0027 (16)
C14A	0.082 (3)	0.053 (2)	0.051 (2)	0.008 (2)	0.006 (2)	0.0042 (19)
C15A	0.108 (4)	0.063 (3)	0.070 (3)	0.009 (3)	0.005 (3)	0.022 (2)
C16A	0.114 (4)	0.078 (3)	0.052 (3)	−0.022 (3)	−0.004 (3)	0.018 (2)
C17A	0.125 (4)	0.079 (3)	0.048 (3)	−0.017 (3)	0.020 (3)	−0.011 (2)
C18A	0.099 (3)	0.051 (2)	0.054 (2)	−0.001 (2)	0.010 (2)	−0.003 (2)
C19A	0.051 (2)	0.056 (2)	0.044 (2)	0.0068 (19)	−0.0024 (17)	−0.0021 (18)
C20A	0.049 (2)	0.075 (3)	0.096 (3)	0.000 (2)	−0.011 (2)	0.009 (3)
C21A	0.057 (3)	0.108 (4)	0.119 (4)	−0.008 (3)	−0.020 (3)	−0.002 (4)
C22A	0.066 (3)	0.130 (5)	0.095 (4)	0.017 (3)	−0.035 (3)	−0.014 (4)
C23A	0.106 (4)	0.111 (5)	0.104 (4)	0.022 (4)	−0.049 (4)	0.019 (4)
C24A	0.085 (3)	0.079 (3)	0.083 (3)	0.001 (3)	−0.029 (3)	0.019 (3)
C25A	0.052 (2)	0.041 (2)	0.048 (2)	−0.0015 (17)	0.0034 (17)	0.0074 (17)
C26A	0.128 (4)	0.057 (3)	0.084 (3)	−0.017 (3)	0.049 (3)	0.004 (2)
C27A	0.130 (5)	0.084 (4)	0.105 (4)	−0.027 (3)	0.056 (4)	0.014 (3)
C28A	0.093 (4)	0.059 (3)	0.112 (4)	−0.024 (3)	0.005 (3)	0.025 (3)
C29A	0.112 (4)	0.053 (3)	0.096 (4)	−0.019 (3)	0.012 (3)	−0.006 (3)
C30A	0.085 (3)	0.054 (3)	0.071 (3)	−0.011 (2)	0.016 (2)	−0.001 (2)
C31A	0.052 (2)	0.043 (2)	0.052 (2)	−0.0026 (17)	0.0153 (18)	0.0024 (17)
C32A	0.062 (3)	0.094 (4)	0.091 (3)	−0.006 (3)	0.014 (2)	−0.038 (3)
C33A	0.099 (4)	0.105 (4)	0.112 (4)	−0.018 (4)	0.031 (4)	−0.063 (3)
C34A	0.094 (4)	0.056 (3)	0.129 (5)	0.005 (3)	0.062 (4)	−0.006 (3)
C35A	0.079 (3)	0.072 (3)	0.096 (4)	0.025 (3)	0.029 (3)	0.015 (3)
C36A	0.064 (3)	0.072 (3)	0.068 (3)	0.023 (2)	0.015 (2)	0.000 (2)
C37A	0.040 (2)	0.0381 (19)	0.052 (2)	0.0045 (16)	−0.0010 (16)	−0.0070 (16)
C38A	0.040 (2)	0.059 (3)	0.172 (5)	0.001 (2)	−0.016 (3)	0.010 (3)
C39A	0.074 (3)	0.038 (2)	0.091 (3)	0.003 (2)	−0.010 (2)	0.010 (2)
Cu1B	0.0353 (2)	0.0377 (2)	0.0452 (2)	−0.00179 (18)	−0.00148 (18)	0.00507 (18)
Cl1B	0.0447 (5)	0.0438 (5)	0.0586 (5)	0.0087 (4)	0.0048 (4)	0.0001 (4)
S1B	0.0314 (4)	0.0413 (5)	0.0698 (6)	−0.0017 (4)	0.0005 (4)	0.0084 (4)
N1B	0.0429 (19)	0.0427 (18)	0.095 (2)	−0.0064 (15)	0.0214 (17)	0.0024 (18)
N2B	0.0422 (18)	0.0444 (18)	0.096 (3)	−0.0026 (15)	0.0175 (17)	−0.0042 (17)
P1B	0.0331 (5)	0.0437 (5)	0.0399 (5)	0.0032 (4)	−0.0007 (4)	0.0026 (4)
P2B	0.0337 (5)	0.0346 (5)	0.0447 (5)	−0.0024 (4)	−0.0023 (4)	0.0050 (4)
C1B	0.0334 (18)	0.053 (2)	0.0374 (18)	0.0036 (16)	0.0004 (14)	0.0003 (16)
C2B	0.042 (2)	0.086 (3)	0.083 (3)	0.010 (2)	−0.003 (2)	0.027 (2)
C3B	0.043 (3)	0.121 (4)	0.099 (4)	0.012 (3)	−0.009 (2)	0.032 (3)
C4B	0.034 (2)	0.116 (4)	0.073 (3)	0.004 (3)	−0.006 (2)	−0.004 (3)
C5B	0.046 (2)	0.084 (3)	0.065 (3)	−0.019 (2)	0.009 (2)	−0.006 (2)
C6B	0.044 (2)	0.062 (2)	0.051 (2)	0.0017 (19)	0.0001 (17)	0.0016 (18)
C7B	0.043 (2)	0.050 (2)	0.0431 (19)	0.0044 (17)	0.0006 (16)	0.0011 (16)
C8B	0.044 (2)	0.088 (3)	0.095 (3)	0.007 (2)	0.004 (2)	−0.029 (3)
C9B	0.057 (3)	0.099 (4)	0.123 (4)	0.021 (3)	0.021 (3)	−0.025 (3)
C10B	0.095 (4)	0.078 (3)	0.076 (3)	0.027 (3)	0.018 (3)	−0.021 (3)
C11B	0.075 (3)	0.075 (3)	0.069 (3)	0.009 (2)	−0.003 (2)	−0.025 (2)
C12B	0.054 (2)	0.073 (3)	0.055 (2)	0.008 (2)	−0.0021 (19)	−0.014 (2)
C13B	0.0369 (19)	0.053 (2)	0.049 (2)	0.0062 (16)	0.0015 (16)	0.0134 (18)
C14B	0.104 (4)	0.061 (3)	0.069 (3)	−0.017 (3)	−0.009 (3)	0.019 (2)
C15B	0.123 (5)	0.066 (3)	0.113 (4)	−0.024 (3)	−0.012 (4)	0.027 (3)
C16B	0.068 (3)	0.087 (4)	0.118 (5)	0.005 (3)	0.016 (3)	0.057 (4)
C17B	0.077 (3)	0.115 (4)	0.070 (3)	0.030 (3)	0.030 (3)	0.049 (3)
C18B	0.067 (3)	0.081 (3)	0.054 (2)	0.014 (2)	0.008 (2)	0.013 (2)
C19B	0.0351 (18)	0.049 (2)	0.048 (2)	−0.0031 (16)	−0.0059 (15)	0.0111 (16)
C20B	0.042 (2)	0.059 (2)	0.082 (3)	0.0007 (19)	0.008 (2)	0.012 (2)
C21B	0.046 (3)	0.084 (3)	0.106 (4)	0.013 (2)	0.015 (2)	0.023 (3)
C22B	0.039 (2)	0.117 (4)	0.098 (4)	−0.005 (3)	0.006 (2)	0.031 (3)
C23B	0.049 (3)	0.095 (4)	0.106 (4)	−0.027 (3)	−0.011 (3)	0.011 (3)
C24B	0.048 (2)	0.061 (3)	0.077 (3)	−0.0116 (19)	−0.004 (2)	0.002 (2)
C25B	0.0345 (18)	0.0389 (19)	0.054 (2)	−0.0018 (15)	−0.0017 (16)	0.0055 (16)
C26B	0.060 (2)	0.046 (2)	0.068 (3)	0.0112 (19)	−0.010 (2)	−0.0017 (19)
C27B	0.066 (3)	0.059 (3)	0.081 (3)	0.007 (2)	−0.013 (2)	−0.021 (2)
C28B	0.053 (3)	0.042 (2)	0.116 (4)	−0.0013 (19)	0.008 (3)	−0.012 (3)
C29B	0.066 (3)	0.047 (2)	0.105 (4)	0.014 (2)	0.007 (3)	0.025 (3)
C30B	0.061 (3)	0.047 (2)	0.068 (3)	0.0073 (19)	0.002 (2)	0.013 (2)
C31B	0.0383 (19)	0.044 (2)	0.0433 (19)	0.0031 (15)	−0.0026 (15)	0.0021 (16)
C32B	0.056 (2)	0.066 (3)	0.051 (2)	−0.013 (2)	−0.0008 (18)	0.0056 (19)
C33B	0.073 (3)	0.081 (3)	0.053 (3)	−0.008 (2)	0.011 (2)	0.010 (2)
C34B	0.069 (3)	0.092 (3)	0.043 (2)	0.012 (3)	−0.009 (2)	−0.003 (2)
C35B	0.053 (2)	0.077 (3)	0.062 (3)	−0.007 (2)	−0.016 (2)	−0.007 (2)
C36B	0.040 (2)	0.062 (2)	0.056 (2)	−0.0023 (18)	−0.0042 (17)	0.0047 (19)
C37B	0.0353 (19)	0.046 (2)	0.050 (2)	−0.0008 (16)	0.0003 (16)	0.0051 (17)
C38B	0.056 (3)	0.065 (3)	0.127 (4)	0.004 (2)	0.041 (3)	0.005 (3)
C39B	0.069 (3)	0.044 (2)	0.149 (5)	−0.007 (2)	0.022 (3)	−0.011 (3)
C1	0.101 (5)	0.227 (8)	0.089 (4)	0.017 (5)	0.022 (4)	0.015 (5)
C2	0.110 (5)	0.123 (5)	0.082 (4)	0.017 (4)	0.016 (4)	0.009 (3)
N3	0.110 (4)	0.152 (5)	0.148 (5)	0.018 (4)	0.002 (4)	0.028 (4)

### Geometric parameters (Å, °)

**Table:**

Cu1A—P1A	2.2847 (9)	Cu1B—Cl1B	2.3956 (9)
Cu1A—P2A	2.2850 (9)	S1B—C37B	1.709 (3)
Cu1A—S1A	2.3715 (10)	N1B—C37B	1.325 (4)
Cu1A—Cl1A	2.4014 (9)	N1B—C38B	1.440 (5)
S1A—C37A	1.709 (3)	N1B—H1BB	0.869 (18)
N1A—C37A	1.331 (4)	N2B—C37B	1.323 (4)
N1A—C38A	1.453 (5)	N2B—C39B	1.451 (5)
N1A—H1AA	0.875 (18)	N2B—H2BB	0.879 (18)
N2A—C37A	1.315 (4)	P1B—C1B	1.833 (3)
N2A—C39A	1.444 (4)	P1B—C7B	1.834 (3)
N2A—H2AA	0.875 (18)	P1B—C13B	1.836 (3)
P1A—C7A	1.832 (3)	P2B—C25B	1.820 (3)
P1A—C13A	1.834 (3)	P2B—C19B	1.834 (3)
P1A—C1A	1.838 (3)	P2B—C31B	1.837 (3)
P2A—C25A	1.825 (3)	C1B—C6B	1.378 (5)
P2A—C19A	1.830 (4)	C1B—C2B	1.380 (5)
P2A—C31A	1.834 (4)	C2B—C3B	1.386 (5)
C1A—C6A	1.358 (5)	C2B—H2B	0.9300
C1A—C2A	1.363 (5)	C3B—C4B	1.354 (6)
C2A—C3A	1.389 (6)	C3B—H3B	0.9300
C2A—H2A	0.9300	C4B—C5B	1.367 (6)
C3A—C4A	1.348 (7)	C4B—H4B	0.9300
C3A—H3A	0.9300	C5B—C6B	1.386 (5)
C4A—C5A	1.349 (7)	C5B—H5B	0.9300
C4A—H4A	0.9300	C6B—H6B	0.9300
C5A—C6A	1.384 (6)	C7B—C12B	1.377 (5)
C5A—H5A	0.9300	C7B—C8B	1.378 (5)
C6A—H6A	0.9300	C8B—C9B	1.378 (6)
C7A—C8A	1.376 (5)	C8B—H8B	0.9300
C7A—C12A	1.378 (5)	C9B—C10B	1.380 (6)
C8A—C9A	1.380 (5)	C9B—H9B	0.9300
C8A—H8A	0.9300	C10B—C11B	1.359 (6)
C9A—C10A	1.348 (6)	C10B—H10B	0.9300
C9A—H9A	0.9300	C11B—C12B	1.368 (5)
C10A—C11A	1.365 (6)	C11B—H11B	0.9300
C10A—H10A	0.9300	C12B—H12B	0.9300
C11A—C12A	1.378 (5)	C13B—C14B	1.363 (5)
C11A—H11A	0.9300	C13B—C18B	1.382 (5)
C12A—H12A	0.9300	C14B—C15B	1.388 (6)
C13A—C18A	1.373 (5)	C14B—H14B	0.9300
C13A—C14A	1.380 (5)	C15B—C16B	1.351 (7)
C14A—C15A	1.386 (5)	C15B—H15B	0.9300
C14A—H14A	0.9300	C16B—C17B	1.349 (7)
C15A—C16A	1.357 (6)	C16B—H16B	0.9300
C15A—H15A	0.9300	C17B—C18B	1.380 (6)
C16A—C17A	1.359 (6)	C17B—H17B	0.9300
C16A—H16A	0.9300	C18B—H18B	0.9300
C17A—C18A	1.386 (6)	C19B—C20B	1.378 (5)
C17A—H17A	0.9300	C19B—C24B	1.381 (5)
C18A—H18A	0.9300	C20B—C21B	1.378 (5)
C19A—C24A	1.368 (5)	C20B—H20B	0.9300
C19A—C20A	1.377 (5)	C21B—C22B	1.357 (6)
C20A—C21A	1.380 (6)	C21B—H21B	0.9300
C20A—H20A	0.9300	C22B—C23B	1.362 (6)
C21A—C22A	1.345 (7)	C22B—H22B	0.9300
C21A—H21A	0.9300	C23B—C24B	1.398 (5)
C22A—C23A	1.341 (7)	C23B—H23B	0.9300
C22A—H22A	0.9300	C24B—H24B	0.9300
C23A—C24A	1.395 (6)	C25B—C30B	1.384 (5)
C23A—H23A	0.9300	C25B—C26B	1.393 (5)
C24A—H24A	0.9300	C26B—C27B	1.374 (5)
C25A—C30A	1.371 (5)	C26B—H26B	0.9300
C25A—C26A	1.379 (5)	C27B—C28B	1.367 (6)
C26A—C27A	1.387 (6)	C27B—H27B	0.9300
C26A—H26A	0.9300	C28B—C29B	1.359 (6)
C27A—C28A	1.339 (7)	C28B—H28B	0.9300
C27A—H27A	0.9300	C29B—C30B	1.387 (5)
C28A—C29A	1.357 (6)	C29B—H29B	0.9300
C28A—H28A	0.9300	C30B—H30B	0.9300
C29A—C30A	1.379 (5)	C31B—C36B	1.378 (5)
C29A—H29A	0.9300	C31B—C32B	1.382 (5)
C30A—H30A	0.9300	C32B—C33B	1.378 (5)
C31A—C32A	1.353 (5)	C32B—H32B	0.9300
C31A—C36A	1.376 (5)	C33B—C34B	1.371 (6)
C32A—C33A	1.396 (6)	C33B—H33B	0.9300
C32A—H32A	0.9300	C34B—C35B	1.370 (5)
C33A—C34A	1.351 (7)	C34B—H34B	0.9300
C33A—H33A	0.9300	C35B—C36B	1.384 (5)
C34A—C35A	1.342 (7)	C35B—H35B	0.9300
C34A—H34A	0.9300	C36B—H36B	0.9300
C35A—C36A	1.378 (6)	C38B—H38A	0.9600
C35A—H35A	0.9300	C38B—H38B	0.9600
C36A—H36A	0.9300	C38B—H38C	0.9600
C38A—H38D	0.9600	C39B—H39D	0.9600
C38A—H38E	0.9600	C39B—H39E	0.9600
C38A—H38F	0.9600	C39B—H39F	0.9600
C39A—H39A	0.9600	C1—C2	1.436 (8)
C39A—H39B	0.9600	C1—H1A	0.9600
C39A—H39C	0.9600	C1—H1B	0.9600
Cu1B—P2B	2.2831 (9)	C1—H1C	0.9600
Cu1B—P1B	2.2989 (9)	C2—N3	1.116 (7)
Cu1B—S1B	2.3857 (9)		
			
P1A—Cu1A—P2A	124.71 (4)	P1B—Cu1B—Cl1B	107.62 (3)
P1A—Cu1A—S1A	107.56 (4)	S1B—Cu1B—Cl1B	108.45 (3)
P2A—Cu1A—S1A	104.04 (4)	C37B—S1B—Cu1B	109.86 (12)
P1A—Cu1A—Cl1A	104.71 (3)	C37B—N1B—C38B	124.9 (3)
P2A—Cu1A—Cl1A	103.01 (3)	C37B—N1B—H1BB	119 (3)
S1A—Cu1A—Cl1A	112.92 (3)	C38B—N1B—H1BB	116 (3)
C37A—S1A—Cu1A	111.93 (12)	C37B—N2B—C39B	124.9 (3)
C37A—N1A—C38A	124.4 (3)	C37B—N2B—H2BB	116 (3)
C37A—N1A—H1AA	120 (3)	C39B—N2B—H2BB	119 (3)
C38A—N1A—H1AA	116 (3)	C1B—P1B—C7B	103.14 (15)
C37A—N2A—C39A	125.5 (3)	C1B—P1B—C13B	102.46 (15)
C37A—N2A—H2AA	114 (2)	C7B—P1B—C13B	101.02 (16)
C39A—N2A—H2AA	120 (2)	C1B—P1B—Cu1B	116.22 (10)
C7A—P1A—C13A	102.24 (15)	C7B—P1B—Cu1B	114.61 (11)
C7A—P1A—C1A	102.89 (15)	C13B—P1B—Cu1B	117.19 (12)
C13A—P1A—C1A	105.53 (16)	C25B—P2B—C19B	102.77 (15)
C7A—P1A—Cu1A	115.08 (11)	C25B—P2B—C31B	105.67 (15)
C13A—P1A—Cu1A	114.40 (11)	C19B—P2B—C31B	99.76 (15)
C1A—P1A—Cu1A	115.16 (11)	C25B—P2B—Cu1B	114.17 (11)
C25A—P2A—C19A	103.78 (17)	C19B—P2B—Cu1B	115.73 (11)
C25A—P2A—C31A	103.71 (16)	C31B—P2B—Cu1B	116.81 (11)
C19A—P2A—C31A	103.47 (17)	C6B—C1B—C2B	118.3 (3)
C25A—P2A—Cu1A	115.18 (12)	C6B—C1B—P1B	118.5 (3)
C19A—P2A—Cu1A	116.67 (12)	C2B—C1B—P1B	123.1 (3)
C31A—P2A—Cu1A	112.51 (12)	C1B—C2B—C3B	120.4 (4)
C6A—C1A—C2A	117.2 (4)	C1B—C2B—H2B	119.8
C6A—C1A—P1A	117.2 (3)	C3B—C2B—H2B	119.8
C2A—C1A—P1A	125.6 (3)	C4B—C3B—C2B	120.8 (4)
C1A—C2A—C3A	120.9 (4)	C4B—C3B—H3B	119.6
C1A—C2A—H2A	119.5	C2B—C3B—H3B	119.6
C3A—C2A—H2A	119.5	C3B—C4B—C5B	119.6 (4)
C4A—C3A—C2A	120.6 (4)	C3B—C4B—H4B	120.2
C4A—C3A—H3A	119.7	C5B—C4B—H4B	120.2
C2A—C3A—H3A	119.7	C4B—C5B—C6B	120.3 (4)
C3A—C4A—C5A	119.5 (4)	C4B—C5B—H5B	119.9
C3A—C4A—H4A	120.3	C6B—C5B—H5B	119.9
C5A—C4A—H4A	120.3	C1B—C6B—C5B	120.6 (4)
C4A—C5A—C6A	119.7 (5)	C1B—C6B—H6B	119.7
C4A—C5A—H5A	120.2	C5B—C6B—H6B	119.7
C6A—C5A—H5A	120.2	C12B—C7B—C8B	117.9 (3)
C1A—C6A—C5A	122.2 (4)	C12B—C7B—P1B	124.8 (3)
C1A—C6A—H6A	118.9	C8B—C7B—P1B	117.2 (3)
C5A—C6A—H6A	118.9	C9B—C8B—C7B	120.6 (4)
C8A—C7A—C12A	116.8 (3)	C9B—C8B—H8B	119.7
C8A—C7A—P1A	118.7 (3)	C7B—C8B—H8B	119.7
C12A—C7A—P1A	124.6 (3)	C8B—C9B—C10B	120.1 (4)
C7A—C8A—C9A	121.6 (4)	C8B—C9B—H9B	119.9
C7A—C8A—H8A	119.2	C10B—C9B—H9B	119.9
C9A—C8A—H8A	119.2	C11B—C10B—C9B	119.6 (4)
C10A—C9A—C8A	120.3 (4)	C11B—C10B—H10B	120.2
C10A—C9A—H9A	119.8	C9B—C10B—H10B	120.2
C8A—C9A—H9A	119.8	C10B—C11B—C12B	120.1 (4)
C9A—C10A—C11A	119.5 (4)	C10B—C11B—H11B	120.0
C9A—C10A—H10A	120.3	C12B—C11B—H11B	120.0
C11A—C10A—H10A	120.3	C11B—C12B—C7B	121.6 (4)
C10A—C11A—C12A	120.2 (4)	C11B—C12B—H12B	119.2
C10A—C11A—H11A	119.9	C7B—C12B—H12B	119.2
C12A—C11A—H11A	119.9	C14B—C13B—C18B	118.0 (4)
C7A—C12A—C11A	121.4 (4)	C14B—C13B—P1B	119.3 (3)
C7A—C12A—H12A	119.3	C18B—C13B—P1B	122.7 (3)
C11A—C12A—H12A	119.3	C13B—C14B—C15B	120.8 (4)
C18A—C13A—C14A	118.2 (3)	C13B—C14B—H14B	119.6
C18A—C13A—P1A	123.6 (3)	C15B—C14B—H14B	119.6
C14A—C13A—P1A	118.2 (3)	C16B—C15B—C14B	120.6 (5)
C13A—C14A—C15A	120.6 (4)	C16B—C15B—H15B	119.7
C13A—C14A—H14A	119.7	C14B—C15B—H15B	119.7
C15A—C14A—H14A	119.7	C17B—C16B—C15B	119.4 (5)
C16A—C15A—C14A	120.1 (4)	C17B—C16B—H16B	120.3
C16A—C15A—H15A	119.9	C15B—C16B—H16B	120.3
C14A—C15A—H15A	119.9	C16B—C17B—C18B	120.9 (5)
C15A—C16A—C17A	120.3 (4)	C16B—C17B—H17B	119.5
C15A—C16A—H16A	119.9	C18B—C17B—H17B	119.5
C17A—C16A—H16A	119.9	C17B—C18B—C13B	120.4 (5)
C16A—C17A—C18A	119.9 (4)	C17B—C18B—H18B	119.8
C16A—C17A—H17A	120.1	C13B—C18B—H18B	119.8
C18A—C17A—H17A	120.1	C20B—C19B—C24B	118.7 (3)
C13A—C18A—C17A	121.0 (4)	C20B—C19B—P2B	117.0 (3)
C13A—C18A—H18A	119.5	C24B—C19B—P2B	124.3 (3)
C17A—C18A—H18A	119.5	C21B—C20B—C19B	121.2 (4)
C24A—C19A—C20A	117.7 (4)	C21B—C20B—H20B	119.4
C24A—C19A—P2A	124.8 (3)	C19B—C20B—H20B	119.4
C20A—C19A—P2A	117.4 (3)	C22B—C21B—C20B	119.7 (4)
C19A—C20A—C21A	120.6 (4)	C22B—C21B—H21B	120.1
C19A—C20A—H20A	119.7	C20B—C21B—H21B	120.1
C21A—C20A—H20A	119.7	C21B—C22B—C23B	120.6 (4)
C22A—C21A—C20A	121.0 (5)	C21B—C22B—H22B	119.7
C22A—C21A—H21A	119.5	C23B—C22B—H22B	119.7
C20A—C21A—H21A	119.5	C22B—C23B—C24B	120.2 (4)
C23A—C22A—C21A	119.3 (5)	C22B—C23B—H23B	119.9
C23A—C22A—H22A	120.3	C24B—C23B—H23B	119.9
C21A—C22A—H22A	120.3	C19B—C24B—C23B	119.6 (4)
C22A—C23A—C24A	120.9 (5)	C19B—C24B—H24B	120.2
C22A—C23A—H23A	119.5	C23B—C24B—H24B	120.2
C24A—C23A—H23A	119.5	C30B—C25B—C26B	117.3 (3)
C19A—C24A—C23A	120.3 (5)	C30B—C25B—P2B	125.8 (3)
C19A—C24A—H24A	119.8	C26B—C25B—P2B	116.9 (3)
C23A—C24A—H24A	119.8	C27B—C26B—C25B	121.4 (4)
C30A—C25A—C26A	117.1 (4)	C27B—C26B—H26B	119.3
C30A—C25A—P2A	118.6 (3)	C25B—C26B—H26B	119.3
C26A—C25A—P2A	124.3 (3)	C28B—C27B—C26B	120.2 (4)
C25A—C26A—C27A	120.1 (4)	C28B—C27B—H27B	119.9
C25A—C26A—H26A	120.0	C26B—C27B—H27B	119.9
C27A—C26A—H26A	120.0	C29B—C28B—C27B	119.7 (4)
C28A—C27A—C26A	121.7 (5)	C29B—C28B—H28B	120.2
C28A—C27A—H27A	119.1	C27B—C28B—H28B	120.2
C26A—C27A—H27A	119.1	C28B—C29B—C30B	120.8 (4)
C27A—C28A—C29A	119.0 (4)	C28B—C29B—H29B	119.6
C27A—C28A—H28A	120.5	C30B—C29B—H29B	119.6
C29A—C28A—H28A	120.5	C25B—C30B—C29B	120.7 (4)
C28A—C29A—C30A	120.2 (5)	C25B—C30B—H30B	119.7
C28A—C29A—H29A	119.9	C29B—C30B—H30B	119.7
C30A—C29A—H29A	119.9	C36B—C31B—C32B	119.0 (3)
C25A—C30A—C29A	121.8 (4)	C36B—C31B—P2B	118.9 (3)
C25A—C30A—H30A	119.1	C32B—C31B—P2B	122.0 (3)
C29A—C30A—H30A	119.1	C33B—C32B—C31B	120.3 (4)
C32A—C31A—C36A	117.7 (4)	C33B—C32B—H32B	119.9
C32A—C31A—P2A	124.7 (3)	C31B—C32B—H32B	119.9
C36A—C31A—P2A	117.5 (3)	C34B—C33B—C32B	120.5 (4)
C31A—C32A—C33A	120.9 (5)	C34B—C33B—H33B	119.8
C31A—C32A—H32A	119.5	C32B—C33B—H33B	119.8
C33A—C32A—H32A	119.5	C35B—C34B—C33B	119.6 (4)
C34A—C33A—C32A	119.8 (5)	C35B—C34B—H34B	120.2
C34A—C33A—H33A	120.1	C33B—C34B—H34B	120.2
C32A—C33A—H33A	120.1	C34B—C35B—C36B	120.3 (4)
C35A—C34A—C33A	120.4 (5)	C34B—C35B—H35B	119.8
C35A—C34A—H34A	119.8	C36B—C35B—H35B	119.8
C33A—C34A—H34A	119.8	C31B—C36B—C35B	120.3 (3)
C34A—C35A—C36A	119.7 (5)	C31B—C36B—H36B	119.9
C34A—C35A—H35A	120.1	C35B—C36B—H36B	119.9
C36A—C35A—H35A	120.1	N2B—C37B—N1B	117.9 (3)
C31A—C36A—C35A	121.5 (4)	N2B—C37B—S1B	120.8 (3)
C31A—C36A—H36A	119.3	N1B—C37B—S1B	121.3 (3)
C35A—C36A—H36A	119.3	N1B—C38B—H38A	109.5
N2A—C37A—N1A	118.5 (3)	N1B—C38B—H38B	109.5
N2A—C37A—S1A	121.2 (3)	H38A—C38B—H38B	109.5
N1A—C37A—S1A	120.3 (3)	N1B—C38B—H38C	109.5
N1A—C38A—H38D	109.5	H38A—C38B—H38C	109.5
N1A—C38A—H38E	109.5	H38B—C38B—H38C	109.5
H38D—C38A—H38E	109.5	N2B—C39B—H39D	109.5
N1A—C38A—H38F	109.5	N2B—C39B—H39E	109.5
H38D—C38A—H38F	109.5	H39D—C39B—H39E	109.5
H38E—C38A—H38F	109.5	N2B—C39B—H39F	109.5
N2A—C39A—H39A	109.5	H39D—C39B—H39F	109.5
N2A—C39A—H39B	109.5	H39E—C39B—H39F	109.5
H39A—C39A—H39B	109.5	C2—C1—H1A	109.5
N2A—C39A—H39C	109.5	C2—C1—H1B	109.5
H39A—C39A—H39C	109.5	H1A—C1—H1B	109.5
H39B—C39A—H39C	109.5	C2—C1—H1C	109.5
P2B—Cu1B—P1B	120.07 (3)	H1A—C1—H1C	109.5
P2B—Cu1B—S1B	106.08 (3)	H1B—C1—H1C	109.5
P1B—Cu1B—S1B	108.79 (3)	N3—C2—C1	178.9 (8)
P2B—Cu1B—Cl1B	105.37 (3)		
			
P1A—Cu1A—S1A—C37A	111.26 (13)	P2B—Cu1B—S1B—C37B	−150.75 (13)
P2A—Cu1A—S1A—C37A	−114.72 (13)	P1B—Cu1B—S1B—C37B	78.79 (13)
Cl1A—Cu1A—S1A—C37A	−3.76 (13)	Cl1B—Cu1B—S1B—C37B	−37.99 (13)
P2A—Cu1A—P1A—C7A	−63.24 (12)	P2B—Cu1B—P1B—C1B	50.41 (13)
S1A—Cu1A—P1A—C7A	58.69 (12)	S1B—Cu1B—P1B—C1B	172.76 (13)
Cl1A—Cu1A—P1A—C7A	179.05 (12)	Cl1B—Cu1B—P1B—C1B	−69.93 (13)
P2A—Cu1A—P1A—C13A	178.77 (12)	P2B—Cu1B—P1B—C7B	−69.88 (13)
S1A—Cu1A—P1A—C13A	−59.29 (12)	S1B—Cu1B—P1B—C7B	52.47 (13)
Cl1A—Cu1A—P1A—C13A	61.07 (12)	Cl1B—Cu1B—P1B—C7B	169.78 (12)
P2A—Cu1A—P1A—C1A	56.23 (13)	P2B—Cu1B—P1B—C13B	171.97 (12)
S1A—Cu1A—P1A—C1A	178.17 (12)	S1B—Cu1B—P1B—C13B	−65.69 (13)
Cl1A—Cu1A—P1A—C1A	−61.48 (13)	Cl1B—Cu1B—P1B—C13B	51.63 (13)
P1A—Cu1A—P2A—C25A	66.15 (14)	P1B—Cu1B—P2B—C25B	66.44 (12)
S1A—Cu1A—P2A—C25A	−57.33 (13)	S1B—Cu1B—P2B—C25B	−57.22 (12)
Cl1A—Cu1A—P2A—C25A	−175.36 (13)	Cl1B—Cu1B—P2B—C25B	−172.10 (12)
P1A—Cu1A—P2A—C19A	−55.91 (14)	P1B—Cu1B—P2B—C19B	−52.59 (13)
S1A—Cu1A—P2A—C19A	−179.39 (13)	S1B—Cu1B—P2B—C19B	−176.25 (13)
Cl1A—Cu1A—P2A—C19A	62.59 (14)	Cl1B—Cu1B—P2B—C19B	68.87 (13)
P1A—Cu1A—P2A—C31A	−175.27 (13)	P1B—Cu1B—P2B—C31B	−169.61 (12)
S1A—Cu1A—P2A—C31A	61.25 (13)	S1B—Cu1B—P2B—C31B	66.73 (12)
Cl1A—Cu1A—P2A—C31A	−56.77 (13)	Cl1B—Cu1B—P2B—C31B	−48.15 (12)
C7A—P1A—C1A—C6A	85.3 (4)	C7B—P1B—C1B—C6B	74.3 (3)
C13A—P1A—C1A—C6A	−167.9 (3)	C13B—P1B—C1B—C6B	178.9 (3)
Cu1A—P1A—C1A—C6A	−40.7 (4)	Cu1B—P1B—C1B—C6B	−52.0 (3)
C7A—P1A—C1A—C2A	−94.5 (4)	C7B—P1B—C1B—C2B	−107.7 (3)
C13A—P1A—C1A—C2A	12.3 (4)	C13B—P1B—C1B—C2B	−3.0 (4)
Cu1A—P1A—C1A—C2A	139.5 (3)	Cu1B—P1B—C1B—C2B	126.1 (3)
C6A—C1A—C2A—C3A	−1.0 (7)	C6B—C1B—C2B—C3B	−1.0 (6)
P1A—C1A—C2A—C3A	178.8 (4)	P1B—C1B—C2B—C3B	−179.1 (3)
C1A—C2A—C3A—C4A	1.1 (8)	C1B—C2B—C3B—C4B	−0.9 (7)
C2A—C3A—C4A—C5A	−0.7 (8)	C2B—C3B—C4B—C5B	2.0 (7)
C3A—C4A—C5A—C6A	0.2 (9)	C3B—C4B—C5B—C6B	−1.1 (7)
C2A—C1A—C6A—C5A	0.5 (8)	C2B—C1B—C6B—C5B	1.9 (5)
P1A—C1A—C6A—C5A	−179.3 (5)	P1B—C1B—C6B—C5B	−180.0 (3)
C4A—C5A—C6A—C1A	−0.1 (9)	C4B—C5B—C6B—C1B	−0.8 (6)
C13A—P1A—C7A—C8A	79.7 (3)	C1B—P1B—C7B—C12B	−4.2 (4)
C1A—P1A—C7A—C8A	−170.9 (3)	C13B—P1B—C7B—C12B	−109.9 (3)
Cu1A—P1A—C7A—C8A	−44.9 (3)	Cu1B—P1B—C7B—C12B	123.1 (3)
C13A—P1A—C7A—C12A	−101.4 (3)	C1B—P1B—C7B—C8B	176.5 (3)
C1A—P1A—C7A—C12A	7.9 (4)	C13B—P1B—C7B—C8B	70.8 (3)
Cu1A—P1A—C7A—C12A	134.0 (3)	Cu1B—P1B—C7B—C8B	−56.2 (3)
C12A—C7A—C8A—C9A	−3.8 (6)	C12B—C7B—C8B—C9B	2.3 (7)
P1A—C7A—C8A—C9A	175.1 (4)	P1B—C7B—C8B—C9B	−178.4 (4)
C7A—C8A—C9A—C10A	0.9 (8)	C7B—C8B—C9B—C10B	−1.7 (8)
C8A—C9A—C10A—C11A	3.0 (8)	C8B—C9B—C10B—C11B	0.1 (8)
C9A—C10A—C11A—C12A	−3.8 (8)	C9B—C10B—C11B—C12B	0.9 (7)
C8A—C7A—C12A—C11A	3.0 (6)	C10B—C11B—C12B—C7B	−0.3 (7)
P1A—C7A—C12A—C11A	−175.9 (4)	C8B—C7B—C12B—C11B	−1.3 (6)
C10A—C11A—C12A—C7A	0.8 (8)	P1B—C7B—C12B—C11B	179.4 (3)
C7A—P1A—C13A—C18A	39.2 (4)	C1B—P1B—C13B—C14B	107.1 (3)
C1A—P1A—C13A—C18A	−68.1 (3)	C7B—P1B—C13B—C14B	−146.7 (3)
Cu1A—P1A—C13A—C18A	164.3 (3)	Cu1B—P1B—C13B—C14B	−21.4 (4)
C7A—P1A—C13A—C14A	−137.6 (3)	C1B—P1B—C13B—C18B	−72.3 (3)
C1A—P1A—C13A—C14A	115.1 (3)	C7B—P1B—C13B—C18B	34.0 (3)
Cu1A—P1A—C13A—C14A	−12.6 (3)	Cu1B—P1B—C13B—C18B	159.3 (3)
C18A—C13A—C14A—C15A	0.6 (6)	C18B—C13B—C14B—C15B	−0.5 (7)
P1A—C13A—C14A—C15A	177.6 (3)	P1B—C13B—C14B—C15B	−179.9 (4)
C13A—C14A—C15A—C16A	0.7 (7)	C13B—C14B—C15B—C16B	1.3 (8)
C14A—C15A—C16A—C17A	−1.0 (8)	C14B—C15B—C16B—C17B	−1.3 (8)
C15A—C16A—C17A—C18A	0.1 (8)	C15B—C16B—C17B—C18B	0.5 (8)
C14A—C13A—C18A—C17A	−1.4 (6)	C16B—C17B—C18B—C13B	0.3 (7)
P1A—C13A—C18A—C17A	−178.3 (3)	C14B—C13B—C18B—C17B	−0.2 (6)
C16A—C17A—C18A—C13A	1.2 (7)	P1B—C13B—C18B—C17B	179.1 (3)
C25A—P2A—C19A—C24A	−3.9 (4)	C25B—P2B—C19B—C20B	−178.2 (3)
C31A—P2A—C19A—C24A	−112.0 (4)	C31B—P2B—C19B—C20B	73.1 (3)
Cu1A—P2A—C19A—C24A	123.9 (3)	Cu1B—P2B—C19B—C20B	−53.1 (3)
C25A—P2A—C19A—C20A	179.9 (3)	C25B—P2B—C19B—C24B	2.4 (3)
C31A—P2A—C19A—C20A	71.9 (3)	C31B—P2B—C19B—C24B	−106.2 (3)
Cu1A—P2A—C19A—C20A	−52.3 (3)	Cu1B—P2B—C19B—C24B	127.5 (3)
C24A—C19A—C20A—C21A	1.9 (7)	C24B—C19B—C20B—C21B	0.4 (6)
P2A—C19A—C20A—C21A	178.4 (4)	P2B—C19B—C20B—C21B	−179.0 (3)
C19A—C20A—C21A—C22A	−2.4 (8)	C19B—C20B—C21B—C22B	−1.6 (7)
C20A—C21A—C22A—C23A	0.9 (9)	C20B—C21B—C22B—C23B	1.9 (7)
C21A—C22A—C23A—C24A	0.9 (9)	C21B—C22B—C23B—C24B	−1.0 (7)
C20A—C19A—C24A—C23A	−0.2 (7)	C20B—C19B—C24B—C23B	0.4 (6)
P2A—C19A—C24A—C23A	−176.3 (4)	P2B—C19B—C24B—C23B	179.8 (3)
C22A—C23A—C24A—C19A	−1.3 (9)	C22B—C23B—C24B—C19B	−0.2 (7)
C19A—P2A—C25A—C30A	91.1 (3)	C19B—P2B—C25B—C30B	−94.0 (3)
C31A—P2A—C25A—C30A	−161.0 (3)	C31B—P2B—C25B—C30B	10.2 (3)
Cu1A—P2A—C25A—C30A	−37.6 (4)	Cu1B—P2B—C25B—C30B	139.9 (3)
C19A—P2A—C25A—C26A	−89.9 (4)	C19B—P2B—C25B—C26B	85.2 (3)
C31A—P2A—C25A—C26A	18.0 (4)	C31B—P2B—C25B—C26B	−170.7 (3)
Cu1A—P2A—C25A—C26A	141.4 (4)	Cu1B—P2B—C25B—C26B	−41.0 (3)
C30A—C25A—C26A—C27A	−0.2 (7)	C30B—C25B—C26B—C27B	1.7 (5)
P2A—C25A—C26A—C27A	−179.2 (4)	P2B—C25B—C26B—C27B	−177.5 (3)
C25A—C26A—C27A—C28A	−2.3 (9)	C25B—C26B—C27B—C28B	−1.6 (6)
C26A—C27A—C28A—C29A	3.4 (9)	C26B—C27B—C28B—C29B	0.2 (6)
C27A—C28A—C29A—C30A	−2.0 (8)	C27B—C28B—C29B—C30B	1.0 (7)
C26A—C25A—C30A—C29A	1.6 (7)	C26B—C25B—C30B—C29B	−0.6 (5)
P2A—C25A—C30A—C29A	−179.3 (4)	P2B—C25B—C30B—C29B	178.5 (3)
C28A—C29A—C30A—C25A	−0.5 (8)	C28B—C29B—C30B—C25B	−0.8 (6)
C25A—P2A—C31A—C32A	−108.7 (4)	C25B—P2B—C31B—C36B	111.2 (3)
C19A—P2A—C31A—C32A	−0.6 (4)	C19B—P2B—C31B—C36B	−142.5 (3)
Cu1A—P2A—C31A—C32A	126.2 (3)	Cu1B—P2B—C31B—C36B	−17.0 (3)
C25A—P2A—C31A—C36A	73.7 (3)	C25B—P2B—C31B—C32B	−71.8 (3)
C19A—P2A—C31A—C36A	−178.2 (3)	C19B—P2B—C31B—C32B	34.5 (3)
Cu1A—P2A—C31A—C36A	−51.4 (3)	Cu1B—P2B—C31B—C32B	160.0 (3)
C36A—C31A—C32A—C33A	−0.3 (7)	C36B—C31B—C32B—C33B	−1.9 (6)
P2A—C31A—C32A—C33A	−177.9 (4)	P2B—C31B—C32B—C33B	−178.9 (3)
C31A—C32A—C33A—C34A	−0.2 (8)	C31B—C32B—C33B—C34B	1.6 (6)
C32A—C33A—C34A—C35A	−0.5 (8)	C32B—C33B—C34B—C35B	−0.3 (7)
C33A—C34A—C35A—C36A	1.7 (8)	C33B—C34B—C35B—C36B	−0.7 (6)
C32A—C31A—C36A—C35A	1.6 (6)	C32B—C31B—C36B—C35B	1.0 (5)
P2A—C31A—C36A—C35A	179.4 (3)	P2B—C31B—C36B—C35B	178.1 (3)
C34A—C35A—C36A—C31A	−2.3 (7)	C34B—C35B—C36B—C31B	0.3 (6)
C39A—N2A—C37A—N1A	5.3 (6)	C39B—N2B—C37B—N1B	5.9 (6)
C39A—N2A—C37A—S1A	−174.0 (3)	C39B—N2B—C37B—S1B	−175.8 (3)
C38A—N1A—C37A—N2A	178.2 (4)	C38B—N1B—C37B—N2B	−178.7 (4)
C38A—N1A—C37A—S1A	−2.5 (6)	C38B—N1B—C37B—S1B	3.0 (6)
Cu1A—S1A—C37A—N2A	−4.3 (3)	Cu1B—S1B—C37B—N2B	27.2 (3)
Cu1A—S1A—C37A—N1A	176.4 (3)	Cu1B—S1B—C37B—N1B	−154.6 (3)

### Hydrogen-bond geometry (Å, °)

**Table:**

*D*—H···*A*	*D*—H	H···*A*	*D*···*A*	*D*—H···*A*
N1A—H1AA···Cl1B^i^	0.88 (2)	2.43 (2)	3.234 (3)	153 (3)
N2A—H2AA···Cl1A	0.88 (2)	2.33 (2)	3.197 (3)	173 (3)
N1B—H1BB···Cl1A	0.87 (2)	2.47 (2)	3.262 (3)	152 (3)
N2B—H2BB···Cl1B	0.88 (2)	2.36 (2)	3.230 (3)	169 (3)

Symmetry codes: (i) *x*+1, *y*, *z*.
